# *Arabidopsis* Serine Decarboxylase Mutants Implicate the Roles of Ethanolamine in Plant Growth and Development

**DOI:** 10.3390/ijms13033176

**Published:** 2012-03-07

**Authors:** Yerim Kwon, Si-in Yu, Hyoungseok Lee, Joung Han Yim, Jian-Kang Zhu, Byeong-ha Lee

**Affiliations:** 1 Department of Life Science, Sogang University, Seoul 121-742, Korea; E-Mails: kyerim@sogang.ac.kr (Y.K.); siin0311@sogang.ac.kr (S.-i.Y.); 2 Division of Life Sciences, Korea Polar Research Institute, Incheon 406-840, Korea; E-Mails: soulaid@kopri.re.kr (H.L.); jhyim@kopri.re.kr (J.H.Y.); 3 Horticulture and Landscape Architecture, Purdue University, West Lafayette, IN 47907, USA; E-Mail: jkzhu@purdue.edu

**Keywords:** serine decarboxylase, ethanolamine, choline, phosphatidylethanolamine, phosphatidylcholine, *Arabidopsis thaliana*

## Abstract

Ethanolamine is important for synthesis of choline, phosphatidylethanolamine (PE) and phosphatidylcholine (PC) in plants. The latter two phospholipids are the major phospholipids in eukaryotic membranes. In plants, ethanolamine is mainly synthesized directly from serine by serine decarboxylase. Serine decarboxylase is unique to plants and was previously shown to have highly specific activity to l-serine. While serine decarboxylase was biochemically characterized, its functions and importance in plants were not biologically elucidated due to the lack of serine decarboxylase mutants. Here we characterized an *Arabidopsis* mutant defective in serine decarboxylase, named *atsdc-1* (*Arabidopsis thaliana serine decarboxylase-1*). The *atsdc-1* mutants showed necrotic lesions in leaves, multiple inflorescences, sterility in flower, and early flowering in short day conditions. These defects were rescued by ethanolamine application to *atsdc-1*, suggesting the roles of ethanolamine as well as serine decarboxylase in plant development. In addition, molecular analysis of serine decarboxylase suggests that *Arabidopsis* serine decarboxylase is cytosol-localized and expressed in all tissue.

## 1. Introduction

**Figure 1:**
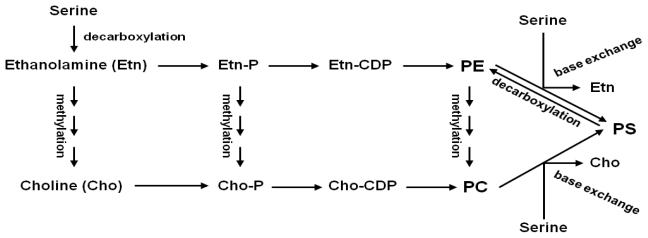
Synthesis of Phosphatidylethanolamine (PE) and Phosphatidylcholine (PC) through the Kennedy Pathway in Plants. Etn-P, phosphoethanolamine; Etn-CDP, cytidine-diphosphoethanolamine; Cho-P, phosphocholine; Cho-CDP, cytidine-diphosphocholine; PE, phosphatidylethanolamine; PC, phosphatidylcholine; PS, phosphatidylserine.

Ethanolamine is an important metabolite for synthesis of phosphatidylethanolamine (PE) and phosphatidylcholine (PC) (Figure 1), which are the two major phospholipids in eukaryotic membranes [1–5]. Unlike most animal cells, ethanolamine can be converted to choline, another precursor for PC synthesis in plant cells [2,4]. Ethanolamine is formed directly or indirectly from serine through direct decarboxylation of serine or base exchange reaction between serine and existing PE (Figure 1) [6–9]. The indirect ethanolamine production by base exchange reaction is found in most eukaryotes including animals and plants, but not fungi [6,9,10]. However, the direct ethanolamine generation by serine decarboxylation is reported only in plants and malarial parasites, *Plasmodium* spp. [7,8,11]. While *Plasmodium* serine decarboxylase gene was not definitively identified [12], plant serine decarboxylase genes were isolated and biochemically shown to be a pyridoxal phosphate-dependent soluble protein with high specific activity to L-serine [7].

Starting from ethanolamine and choline, de novo biosynthesis of PE and PC in most eukaryotic cells occurs through the “Kennedy” pathway, consisting of two parallel CDP-ethanolamine and CDP-choline pathways (Figure 1) [1,13]. PE can also be synthesized via the decarboxylation of phosphatidylserine (PS) by PS decarboxylases that is pyruvoyl cofactor-dependent and membrane-localized [9]. PC can be formed from PE through three successive methylations by PE *N*-methyltransferases (PEMTs). In plants, the methylation of ethanolamine moiety to form choline moiety by *N*-methyltransferases can occur at the levels of free ethanolamine bases, phospho-ethanolamine, and PE [4], linking two CDP-ethanolamine and CDP-choline pathways. PE and PC not only function in maintaining eukaryotic membrane structures, but also contribute to the pools of many biologically active molecules such as phosphatidic acid and diacylglycerol that act as signal molecules [1].

Through analyses of *Arabidopsis* mutants’ phenotypes and identification of the gene responsible for the phenotypes, the functions of many genes in *Arabidopsis* have been elucidated. Indeed, many functions of membrane lipids have been uncovered through many *Arabidopsis* lipid defective mutants [14]. For example, when phosphoethanoleamine *N*-methyltransferase, one enzyme that convert ethanolamine moiety to choline moiety, was silenced, the *Arabidopsis* mutant displayed pale-green leaves, early senescence, temperature-sensitive male sterility and salt hypersensitivity, revealing the importance of choline biosynthesis in plant growth and stress responses [15].

Although ethanolamine is thought to be synthesized mainly via serine decarboxylation [7,8], our understanding on the biological importance of ethanolamine is limited due to the lack of serine decarboxylase mutants.

Here we report the characterization of an *Arabidopsis atsdc-1* (*Arabidopsis thaliana serine decarboxylase-1*) mutant defective in serine decarboxylase. Our analysis suggests that serine decarboxylase is important in maintenance of leaf tissue, lateral inflorescence development, floral fertility and flowering regulation in short days. Moreover, we found that serine decarboxylase is cytosol-localized and expressed in all tissue.

## 2. Results

### 2.1. Atsdc-1 Developmental Defects and Their Rescue under Short Day Conditions

One *Arabidopsis* mutant with growth defects was isolated from an ethyl methanesulfonate (EMS)-mutagenized *Arabidopsis* pool and later named *atsdc-1* (*Arabidopsis thaliana serine decarboxylase-1*) (see below for the phenotypes and the reason for naming).

**Figure 2:**
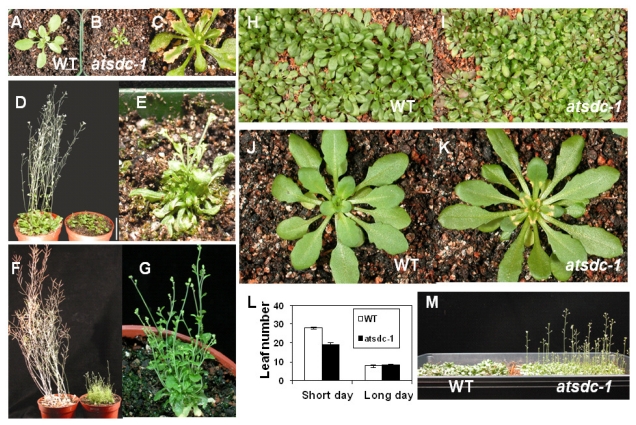
Growth and Development of *atsdc-1.* (**A**–**C**): 3-week-old wild type (**A**) and *atsdc-1* (**B** and **C**) grown under long day conditions. Bar = 1 cm. **C** is a magnified image of **B. (D** and **E)**: 5-week-old wild type (left in **D**) and *atsdc-1* (right in **D**) grown under long day conditions. Bar = 5 cm. **E** is a magnified image of one of the *atsdc-1* seedlings. (**F** and **G)**: 7-week-old wild type (left in **F**) and *atsdc-1* (right in **F**) grown under long day conditions. **G** is a magnified image of one of the *atsdc-1* seedlings. (**H** and **I**): 38-day-old wild type (**H**) and *atsdc-1* (**I**) grown under short day conditions. (**J** and **K**): 43-day-old wild type (**J**) and *atsdc-1* (**K**) grown under short day conditions. (**L**): Comparisons of leaf number upon bolting between the wild type and *atsdc-1*. (error bars = standard error; *n* = 20) (**M**): Difference in bolting between the wild type and *atsdc-1* under short day conditions (63-day-old).

Under long day conditions, the size of *atsdc-1* seedlings was much smaller than that of the wild type and the mutant never reached the height of the mature wild type (Figure 2A–G). Necrosis was observed along the edges of the mutant leaves (Figure 2C). *atsdc-1* was sterile and had multiple inflorescences (Figure 2G). Interestingly, the smaller size and multiple inflorescence defects in the mutant could be rescued under short day conditions (Figure 2H–K,M). Nevertheless, the mutant leaves under short day conditions still showed necrosis along the edges, although its appearance was delayed (Figure 2J,K). Under short day conditions, *atsdc-1* flowered earlier than the wild type; the mutant bolted at an average leaf number of 19, whereas the wild type bolted at an average leaf number of 28 (Figure 2L,M). Under long day conditions, there was no difference in flowering time between the wild type and *atsdc-1*; both bolted at an average leaf number of 8 (Figure 2L).

### 2.2. Molecular Cloning of AtSDC

None of the 25 F1 plants from a cross between the wild type and *atsdc-1* showed the *atsdc-1* morphological phenotypes. In the successive F2 generation, 403 seedlings tested were found to segregate into 292 wild-type phenotypes and 111 *atsdc-1* phenotypes (3:1, χ^2^= 0.0042, *p* = 0.837). Taken together, these results suggest that *atsdc-1* is a single recessive nuclear mutation.

**Figure 3:**
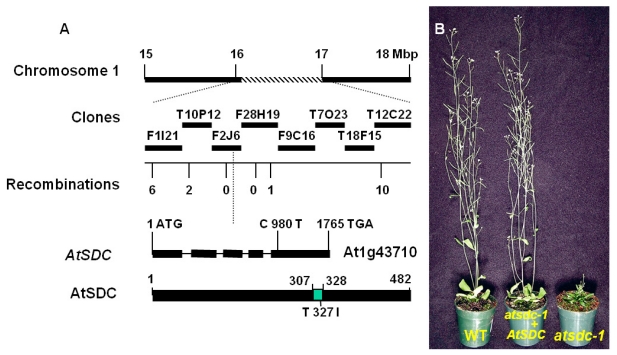
Molecular Cloning and Complementation of *atsdc-1*. (**A**): Map-based cloning of *AtSDC*. Simple sequence length polymorphism (SSLP) markers used were, from left to right above the recombination mark; F1I21-6K, T10P12-20K, F2J6-92K, F28H19-10K, F28H19-83K, and T12C22-11K. The number of recombination events was out of a total of 948 chromosomes. The mutation in *AtSDC* gene is a C to T change at 980 nt, which resulted in threonine to isoleucine change at the 327th amino acid. Green box indicates the PLP binding domain; (**B**): Molecular complementation of *atsdc-1* developmental defects in 5-week-old *atsdc-1* transformed with the wild type *AtSDC* gene (T1).

To identify the *AtSDC* gene responsible for the *atsdc-1* phenotypes, we performed map-based cloning of the *atsdc-1* mutant gene. We selected the *atsdc-1* mutants in the F2 mapping population from a cross between Ler and *atsdc-1*. Initial mapping delimited the mutation to the middle of chromosome 1, between the nga248 and nga280 simple sequence length polymorphism (SSLP) markers. For fine mapping, new SSLP markers were developed, and the mutation was narrowed down to the F2J6 and F28H19 BAC clones (Figure 3A). Through sequencing genes in this region and comparing the wild type and *atsdc-1* sequences, a mutation was found in the At1g43710 gene. The mutation was a change of C to T that took place at 980th nucleotide from the putative initiation codon of At1g43710 (Figure 3A). The mutation resulted in an alteration of the polar amino acid threonine to the nonpolar amino acid isoleucine at the putative 327th amino acid (Figure 3A).

To confirm the authenticity of the gene, a genomic DNA fragment that spans from 1276 base pair 5′ upstream of the initiation codon to 410 base pair 3′ downstream of the stop codon of the At1g43710 open reading frame was subcloned into a binary vector and transferred into the *atsdc-1* mutant through *Agrobacterium*-mediated transformation. The resulting T1 transformants did not exhibit the *atsdc-1* developmental defects (Figure 3B). These results confirmed that At1g43710 is indeed *AtSDC*, and the *atsdc-1* mutation is responsible for the *atsdc-1* morphological defects.

### 2.3. AtSDC Encodes a Serine Decarboxylase

Results of BLAST searches of the *Arabidopsis* genome database suggested that *AtSDC* is a single-copy gene in the *Arabidopsis* genome. An *AtSDC* cDNA with a complete coding sequence was available from the *Arabidopsis* Biological Resource Center under the clone id U09195 [16]. Comparison of the genomic and cDNA sequences suggested that the *AtSDC* gene contains 5 exons and 4 introns with a 1449-bp coding sequence that would result in a 482-amino acid protein. The AtSDC protein did not have any detectable cellular targeting domains. It contains a putative pyridoxal phosphate (PLP) binding site spanning from 307 to 328 amino acids (Figure 3A). The PLP binding site is a conserved domain among many PLP-dependent decarboxylases, including glutamate-, histidine-, tryptophan-, and tyrosine-decarboxylases [17]. The *atsdc-1* mutation occurred in the putative PLP binding site (Figure 3A). Therefore, the mutation might affect PLP binding to the AtSDC protein. *AtSDC* was annotated in the *Arabidopsis* database [18] as a histidine decarboxylase. However, Rotein *et al.* (2001) showed that *AtSDC* is in fact a serine decarboxylase. The authors expressed *Arabidopsis* AtSDC recombinant protein in *E. coli* using *AtSDC* cDNA and found specific decarboxylase activity on l-serine. They also showed that AtSDC does not have significant activity in histidine decarboxylation.

**Figure 4:**
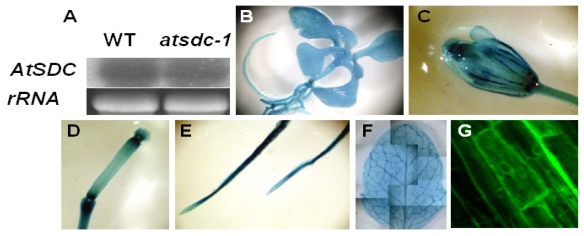
Expression Patterns of *AtSDC* and *AtSDCp*-*GUS,* and Localization of GFP-AtSDC Fusion Protein. (**A**): *AtSDC* expression in the wild type and *atsdc-1*. rRNA gel picture was shown as loading control (**B**–**F**): *AtSDC* promoter-driven GUS activity in the whole seedling (**B**), flower (**C**), silique (**D**), roots (**E**) and leaf (**F**). (**G**): Subcellular localization of the GFP-AtSDC fusion protein in root cells.

We obtained an *Arabidopsis* line (SALK_070968) with a T-DNA insertion [19] in the second intron of *AtSDC*. Through PCR genotyping out of 12 plants from this line (data not shown), we isolated two plants heterozygous for the T-DNA insertion. In the generation following self-pollination of the two heterozygous plants, we still could not identify any plants that were homozygous for the T-DNA insertion (data not shown). This suggested that the T-DNA insertion (potentially full knockout of function) in *AtSDC* is lethal, and our *atsdc-1* EMS mutant is a weak allele. In support of the essential role of *AtSDC* in plant viability, another *atsdc-1* T-DNA allele discovered by the Seed Gene Project [20] is a recessive-embryo-defective mutant (hence named *embryo defective 1075*), and the germplasm (ABRC id: CS16096) is maintained as a heterozygote [21]. RNA blot analysis showed that the *AtSDC* transcript level was not affected in *atsdc-1* (Figure 4A), suggesting that the *atsdc-1* mutation does not affect the steady state level of the transcript.

To study the expression pattern of *AtSDC*, we investigated its promoter activity by fusing an 1126-bp genomic DNA fragment upstream of the *AtSDC* ORF to the β-glucuronidase (*GUS*) reporter gene (*AtSDCp*-*GUS*). Transgenic *Arabidopsis* plants expressing the *AtSDCp*-*GUS* reporter were then subjected to histochemical GUS analysis. GUS activity was detected in whole seedlings and in all organs tested from adult plants, including flowers, sliques, roots, and leaves (Figure 4B–F). This result suggests a ubiquitous expression of *AtSDC*.

Transgenic *Arabidopsis* plants expressing the GFP-AtSDC fusion protein were generated to study AtSDC subcellular localization. Confocal microscopy analysis with the roots of the *GFP-AtSDC* transgenic *Arabidopsis* detected green fluorescence in the cytosol and plasma membrane, suggesting a cytosolic and plasma membrane localization of the AtSDC protein (Figures 4G). However, database searches showed no detabtable transmembrane domains in AtSDC. Also, some known cytosolic proteins displayed peripheral fluorescence when fused with GFP [22]. Although further analysis will clarify the exact AtSDC subcellular localization, it is likely that AtSDC is cytosolic localized.

### 2.4. Ethanolamine Rescues the atsdc-1 Developmental Defects

**Figure 5:**
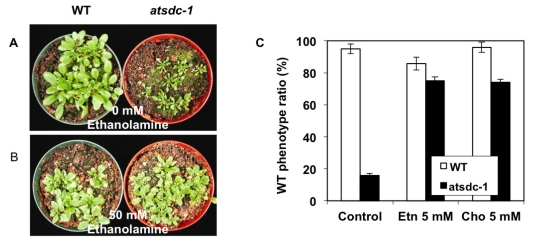

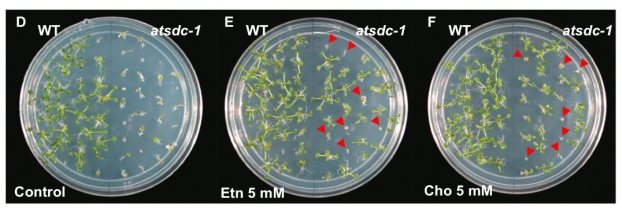
*atsdc-1* Morphology After Ethanolamine or Choline Treatment. (**A-B**): Comparison of the wild type and *atsdc-1* with or without 50 mM ethanolamine treatment. (**C**): *atsdc-1* morphology recovery with ethanolamine (Etn) or choline (Cho) treatment in MS agar plates. WT phenotype ratios were obtained by dividing numbers of normally grown WT seedlings divided with total germinated seeds. Results are from three replicates (error bar = standard error). (**D-F**): Comparison of the wild type and *atsdc-1* on MS agar plates supplemented with or without 5 mM ethanolamine or 5 mM choline. Red arrow heads indicate unrecovered seedlings. Small recovered *atsdc-1* seedlings on ethanolamine and choline plates were eventually grown to the wild type size.

Because AtSDC is involved in the conversion of serine to ethanolamine and the eventual biosynthesis of choline [7], the *atsdc-1* mutant seedlings should be deficient in these metabolites. We therefore applied ethanolamine or choline to *atsdc-1* mutant plants growing in soil or MS agar plates. Although the application of 50 mM ethanolamine on soil grown plants had only a slight effect on the growth of the wild type, it remarkably rescued the *atsdc-1* developmental defects (Figure 5A,B).

It also rescued *atsdc-1* sterility (data not shown). Similarly, *atsdc-1* morphology recovered almost to that of the wild-type in MS agar plates with ethanolamine or choline (Figure 5C–F). Although choline application also rescued the defects in the *atsdc-1* mutant, the effect was not as efficient on mutant plants grown in soil as compared with those on MS plates (data not shown). The rescue by ethanolamine or choline suggests that the *atsdc-1* mutant is indeed defective in the conversion of serine to ethanolamine and might accumulate lower amounts of these metabolites than the wild type.

## 3. Discussion

In this study, we used a forward genetics screen to identify a locus, *AtSDC*, that encodes a serine decarboxylase and is crucial for plant growth. Serine decarboxylase is involved in the direct conversion of serine to ethanolamine [7]. Plants form ethanolamine through either serine decarboxylation or base exchange reaction of PS, but serine decarboxylation is thought to be the major source of ethanolamine in plants [7,8]. Ethanolamine is an important metabolite in plants for the synthesis of choline and membrane lipids, such as PE and PC, as well as choline betaine in some species. Thus, a deficiency in these ethanolamine-derived metabolites might undermine the membrane system. Many lipid-related *Arabidopsis* mutants display varying degrees of abnormal morphological phenotypes, which indicates a crucial role of lipid biosynthesis in plant growth and development [14]. Indeed, many *Arabidopsis* mutants impaired in functions of genes involved in the Kennedy pathway displayed abnormal growth and development. PE N-methyltransferase (PEMT)-silenced *Arabidopsis* mutant (*t365*) plants were pale green, early aging, and exhibited temperature-sensitive male sterility [15]. Mutations in CTP:PE cytidylyltransferase caused embryo abortion, dwarfism, and reduced fertility in *Arabidopsis* [23]. Defects in a base exchange type PS synthase also resulted in embryo lethality and sterile dwarfism [24]. These abnormalities were also observed in *atsdc-1* confirming the important functions of ethanolamine-derived metabolites in these developmental processes.

The cytosolic localization of GFP-AtSDC is consistent with the soluble property of AtSDC protein [7]. The ubiquitous *AtSDCp*-*GUS* activity is also in agreement with the universal requirement of serine decarboxylase in membrane lipid biosynthesis. The defect in serine decarboxylase in *atsdc-1* was confirmed by the rescue of *atsdc-1* morphological defects by ethanolamine (Figure 5) or choline application. Thus, it appears that the *atsdc-1* defects are due to the deficiency of ethanolamine and/or ethanolamine-derived metabolites in *atsdc-1*.

The nearly normal growth and development in *atsdc-1* under short day conditions is intriguing. Apparently, the ethanolamine requirement is either low, or other compensation pathways might become activated under short day conditions. An example of a conditional phenotype is that found in the *fab2 Arabidopsis* mutant, which is defective in the desaturation of stearic acid (18:0) to oleic acid (18:1) [25]. The *fab2* mutation results in an elevated level of stearic acid in membrane lipids. At 22 °C, *fab2* shows a dramatic dwarf phenotype, which can be restored to a normal phenotype with high temperatures (*i.e.*, 36 °C) [25]. This phenotype recovery did not include changes in fatty acid composition. Thus, this recovery was proposed to be due to the increased fluidity of lipids with stearic acid at high temperatures. *pect1-4*, defective in CTP:PE cytidylyltransferase and PEMT-silenced *Arabidopsis* were also shown to display temperature-sensitive developmental defects [15,20].

The *atsdc-1* growth temperature was set at 22 °C under both long and short day conditions. Therefore, it appears that the different photoperiods somehow brought about metabolic differences in plants. Since plants are photosynthetic organisms, it is not difficult to hypothesize that their metabolism depends on the duration of light illumination. The induction of different genes and physiological changes under different photoperiods are well documented [26–28]. Interestingly, the early bolting phenotype of *atsdc-1* was observed only under short day conditions. This observation suggests that short day inhibition of flowering may require metabolism that involves proper AtSDC activity and probably ethanolamine-derived metabolites in plants.

## 4. Experimental Section

### 4.1. Plant Materials and Growth Conditions

*Arabidopsis thaliana* Columbia *gl1* plants were mutated by use of ethyl methanesulfonate to generate the mutant seed pool (M2 generation). For *Arabidopsis* cultivation, *Arabidopsis* seeds were put on soil or MS (Murashige and Skoog salt base, JRH Biosciences, Lenexa, KS) agar (0.6%) plates supplemented with 3% sucrose and placed at room temperature (22 ± 1 °C) under continuous light after 2–3 day cold stratification. When necessary, seedlings on MS were transferred to soil pots in a growth chamber under 22 °C cycles of 16-h light/8-h dark. For short day conditions, 22 °C cycles of 8-h light/16-h dark were used. Stock solutions of 1 M ethanolamine and choline chloride (Sigma, St. Louis, MO, USA) were prepared, and the pH was adjusted to 5.8 with HCl when necessary. Appropriate working concentrations were then prepared. Ethanolamine or choline was added into the MS agar media or sprayed every 4 to 5 days on soil-grown *Arabidopsis* plants.

### 4.2. Gene Expression Analysis

Nine-day-old seedlings grown on MS agar plates were used for RNA analysis. Total RNA was extracted and analyzed as described previously [29]. Briefly, total RNA was extracted with extraction buffer containing 50 mM Tris-HC1, pH 8.0, 300 mM NaCl, 5 mM EDTA, 2% SDS, 2 mM aurintricarboxylic acid, and 10 mM beta-mercaptoethanol. Extracted total RNA was separated on formaldehyde-agarose gel and the resulting gel was blotted onto nylon membrane. The membrane was then hybridized with ^32^P labeled DNA fragment probe of the specific genes. As a loading control, the β-tubulin gene was amplified by PCR with the following primer pairs: Tubulin-F (5′-CGTGGATCACAGCAATACAGAGCC-3′) and Tubulin-R (5′-CCTCCTGCACTTCCACTTCGTCTTC-3′). For the *AtSDC* probe, *AtSDC* full-length cDNA was used. After washing, the membrane was exposed to X-ray film.

### 4.3. Positional Cloning

For genetic mapping of the *atsdc-1* mutation, *atsdc-1* was crossed with the wild type in the ecotype Landsberg *erecta*. The resulting F1 plants were allowed to self and the F2 seeds collected. Homozygous *atsdc-1* mutations in the segregated F2 population were selected on the basis of their developmental defects. The mutation was mapped with use of SSLP markers [30]. If necessary, new SSLP markers were developed by use of the Monsanto *Arabidopsis* Polymorphism collection [31]. Newly developed SSLP markers are as follows: F1I21-6K (F1I21-6KF, 5′-TCCAGTCCTT AAGCGGATTT-3′; F1I21-6KR, 5′-GCATAACACAATGTTCAGACAAA-3′), T10P12-20K (T10P12-20KF, 5′-TAGCAAAGCTTTCGATCCAT-3′; T10P12-20KR, 5′-ATTCTGTTGGGTTG CTATGC-3′), F2J6-92K (F2J6-92KF, 5′-GTGCGGGAGTGTGATAGAAT-3′; F2J6-92KR, 5′-TCC TCGAAAGATTCATTGATTT-3′), F28H19-10K (F28H19-10KF, 5′-GTGCGGGAGTGTGAT AGAAT-3′; F28H19-10KR, 5′-TCCTCGAAAGATTCATTGATTT-3′), F28H19-83K (F28H19-83KF, 5′-GGAGCCAAAACCAGCAACTA-3′; F28H19-83KR, 5′-GTTCTTGGTTGAGTGGAACG-3′), T12C22-11K (T12C22-11KF, 5′-TCCACCGGAGTAAACTCCATA-3′; T12C22-11KR, 5′-TCCG GAGATAGCTCAACAGC-3′).

### 4.4. Plasmid Construction and Plant Transformation

The F2J6 BAC clone and U09195 cDNA clone were obtained from the *Arabidopsis* Biological Resource Center (Columbus, OH, USA) and used as a PCR template for *AtSDC* genomic DNA or ORF amplification. For *atsdc-1* complementation, the 3451-bp genomic DNA fragment of *AtSDC* covering 1276 bp upstream of the translation start codon to 410 bp downstream of the stop codon was amplified by LA taq polymerase (Takara Shuzo CO., Shiga, Japan) with use of F2J6 BAC DNA as a template and the following primers: L126GKpnI-F (5′-CGGGGTACCT CATGTTCTCCGAGAGTTAGGGATA-3′) and L126GXbaI-R2 (5′-GCTCTAGACTTTTGTGT TGTTAGCGTCTATGCAG-3′). The resulting fragment was cloned into pCAMBIA1200 between the KpnI and XbaI sites, resulting in pCAM1200-*AtSDCg*. For the *AtSDC* promoter-driven *GUS* (β-glucuronidase) construct, a 1126-bp fragment spanning −1134~−9 upstream of the *AtSDC* ORF was amplified by PCR with use of F2J6 BAC DNA as a template and the primer pair L126pEcoRI-F (5′-GAAAGAATTCCTTTGGGGTCTTTGGTTG-3′) and L126pBamHI-R (5′-TAAAGGATCCTGT AGATCTGAGTGATTGGA-3′). The *AtSDC* promoter fragment was then subcloned into pCAMBIA1381 between the EcoRI and BamHI sites, which resulted in pCAM1381-*AtSDCp-GUS*. For the construct for the GFP-AtSDC fusion protein, *AtSDC* ORF was amplified by PCR with U09195 cDNA used as a template and the primer pair L126GFP-XhoI-F (5′-TTAAACTCGAGAT GGTTGGATCTTTGGAATCTG-3′) and L126GFP-BamHI-R (5′-AATGGGGATCCCGCTTGT GAGCTGGACAGAT-3′). The amplified *AtSDC* ORF was subcloned into pEZTNL between the XhoI and BamHI sites, which resulted in pEZTNL-*GFP-AtSDC*. pCAM1200-*AtSDCg* and pCAM1381-*AtSDCp-GUS* were transferred to *Agrobacterium* GV3101 (pMP90) and pEZTNL-*GFP-AtSDC* to *Agrobacterium* LBA4404 by electroporation at 1250V with capacitance of 25 F and resistance of 400. After appropriate antibiotic selection and PCR confirmation, selected *Agrobacterium* was grown at 28 C in LB medium (Luria-Bertani, bacto-tryptone 1% (w/v), bacto-yeast extract 0.5% (w/v), NaCl 1% (w/v) pH 7.0) overnight and then used for *in planta* transformation by floral infiltration.

### 4.5. Microscopic Analysis

Glufosinate-ammonium resistant *GFP-AtSDC* transgenic seedlings, selected in soil by spraying 30 mg/L Finale (AgrEvo Environmental Health, Montvale, NJ, USA), were mounted on glass slides, and green fluorescence images were taken under a BioRad MRC-1024 confocal laser-scanning microscope with 488 nm laser excitation and an emission filter of 522/DF35. For GUS staining, the buffer 3 mM X-Gluc, 0.1 M Na-phosphate buffer, pH 7, 0.1% Triton X-100, and 8 mM β-mercaptoethanol was added to the *AtSDCp*-*GUS* transgenic seedlings. The samples were incubated overnight at 37 °C and subjected to treatment with 70% ethanol at 70 °C to remove chlorophyll. GUS pictures were taken under a dissecting microscope.

## 5. Conclusions

In this study, we have characterized the *Arabidopsis atsdc-1* mutant defective in serine decarboxylase and analyzed the expression of the *AtSDC* gene. The *atsdc-1* mutants showed leaf necrosis, multiple inflorescence development, floral sterility, and early flowering in short day conditions. These defects were rescued by ethanolamine or choline application to *atsdc-1*, implicating that ethanolamine and ethanolamine-derived metabolites are important in these developmental processes. Gene expression analysis of the *AtSDC* gene suggests that *Arabidopsis* serine decarboxylase is cytosol-localized and expressed in all tissue.
